# In Vivo and In Vitro Models of Hepatic Fibrosis for Pharmacodynamic Evaluation and Pathology Exploration

**DOI:** 10.3390/ijms26020696

**Published:** 2025-01-15

**Authors:** Yanting Hu, Zhongrui Zhang, Akida Adiham, Hong Li, Jian Gu, Puyang Gong

**Affiliations:** College of Pharmacy and Food, Southwest Minzu University, Chengdu 610093, China; h18185425548@163.com (Y.H.); 18715911023@163.com (Z.Z.); akida102800@163.com (A.A.); lh9912250114@163.com (H.L.)

**Keywords:** hepatic fibrosis, hepatic stellate cells, animal model, drug screening

## Abstract

Hepatic fibrosis (HF) is an important pathological state in the progression of chronic liver disease to end-stage liver disease and is usually triggered by alcohol, nonalcoholic fatty liver, chronic hepatitis viruses, autoimmune hepatitis (AIH), or cholestatic liver disease. Research on novel therapies has become a hot topic due to the reversibility of HF. Research into the molecular mechanisms of the pathology of HF and potential drug screening relies on reliable and rational biological models, mainly including animals and cells. Hence, a number of modeling approaches have been attempted based on human dietary, pathological, and physiological factors in the development of HF. In this review, classical and novel methods of modeling HF in the last 10 years were collected from electronic databases, including Web of Science, PubMed, ScienceDirect, ResearchGate, Baidu Scholar, and CNKI. Animal models of HF are usually induced by chemical toxicants, special diets, pathogenic microorganisms, surgical operations, and gene editing. The advantages and limitations of hepatic stellate cells (HSCs), organoids, and 3D coculture-based HF modeling methods established in vitro were also proposed and summarized. This information provides a scientific basis for the discovery of the pathological mechanism and treatment of HF.

## 1. Introduction

Hepatic fibrosis (HF) is a crucial pathological condition that occurs in many chronic liver diseases and is caused by persistent liver injury and the formation of fibrous scarring. HF is characterized by excessive deposition of the extracellular matrix (ECM) formed from crosslinked collagens (type I and type III), fibronectin, elastin fibers, glycoproteins, and mucopolysaccharides, which disrupts liver structure and function, ultimately leading to liver failure [1,2]. If not effectively intervened, HF can progress to cirrhosis or even hepatocellular carcinoma (HCC), resulting in high mortality and medical burdens [3,4]. Hence, novel therapeutic strategies to reverse HF have become a research hotspot in the field of hepatopathy. Hepatic stellate cells (HSCs) are a major component of hepatic non-parenchymal cells; when stimulated with various hepatic injury factors, activated HSCs (aHSCs) transform into myofibroblasts (MFBs), which leads to the deposition of the ECM and the progression of HF [5,6]. However, HF can be reversed by reducing pathogenic factors of chronic liver injury, inhibiting the activation of HSCs, increasing collagenase activity, and degrading the ECM [7]. The proliferation and activation of MFBs, the main source of the ECM, play vital roles in the fibrotic process. HSCs are the main source of these ECM-producing MFBs, which are found only in the injured liver [5,6]. However, HF can be reversed by reducing factors associated with chronic liver injury, eliminating MFBs and fibrotic scars, increasing collagenase activity, and degrading the ECM [7]. Owing to the diverse causes and complex pathologic processes of HF, appropriate in vivo and in vitro models are highly important for obtaining an in-depth understanding of the nature of the disease and drug development.

Currently, many in vivo animal models have been used for studies of anti-HF drugs, and a number of in vitro modeling approaches have emerged. Each modeling method has a specific induction method, dosage, timing, and other factors to simulate the characteristics of HF under different inducements. Hence, choosing a more appropriate model in the face of differences in the degree of fibrosis and the symptoms of different liver diseases is particularly important. Therefore, this paper reviews the commonly used in vivo and in vitro HF models, the progress of advanced models, and the development of composite models. The principles, approaches, characteristics, and pros and cons of various models are summarized, and the similarities between the symptoms of each HF model and human symptoms are also elucidated, with the aim of providing a more comprehensive basis for selecting relevant models and providing more references for targeted research on HF.

## 2. In Vivo Hf Models

### 2.1. HF Animal Models Induced by Chemicals

The most used in vivo model of HF is induced by chemicals, mainly including ethanol, carbon tetrachloride (CCl_4_), thioacetamide (TAA), and dimethylnitrosamine (DMN). Animals receive chemical poisons by oral or intraperitoneal (IP) injection to simulate the distinctive process of HF over a much shorter period. Chemical molding methods also have the advantages of simple operation and high reproducibility.

#### 2.1.1. HF Animal Model Induced by Ethanol

Alcohol is a predisposing factor for numerous chronic liver diseases, and excessive alcohol consumption leads to fatty degeneration of the liver and the development of alcohol-related liver disease (ALD), which can progress to HF and cirrhosis [8]. In the liver, hepatocytes, which make up 70% of the liver mass, constitute the core region of ethanol metabolism. Excess ethanol increases the generation of metabolic enzymes for oxidative metabolism via three pathways, alcohol dehydrogenase, cytochrome P4502E1 (CYP2E1) (the main inducible pathway), and catalase, which lead to increases in the levels of cytotoxic acetaldehyde and deleterious pro-oxidant agents [9]. Owing to the complicated effects of ethanol in liver cells, inflammatory signs of chronic liver disease occur after the replacement of the liver parenchyma by nonfunctional connective tissue, which manifests itself as a progression to cirrhosis [10]. Feeding mice or rats with alcohol by voluntary ingestion or intragastric feeding tubes can result in only mild hepatic injury, which is largely unable to develop into the later stages of fibrosis [11]. With the later emergence of more alcohol-based models, such as the Lieber–DeCarli liquid diet model, the Tsukamoto–French (TF) model, and others, hepatic steatosis or more severe hepatic injury can be observed, but it still does not imitate the degree of HF that occurs in human disease or requires a “second hit” to achieve it [12]. Moreover, the risk of advanced fibrosis is highest in patients with type 2 diabetes mellitus (T2DM) who consume moderate amounts of alcohol, confirming the synergistic role of alcohol in the profibrotic process [13]. Therefore, to overcome these limitations, ethanol should be combined with specific diets, and chemicals such as CCl_4_, viral infections, and genetically inherited mutations may induce significant HF features in animals.

#### 2.1.2. HF Animal Model Induced by Carbon Tetrachloride (CCl_4_)

CCl_4_ is a type of polychlorinated hydrocarbon that is highly toxic to the kidney, testicles, brain, heart, lung, and other tissues, especially the liver [14]. The hepatotoxicity of CCl_4_ can destroy the function of liver cells and affect the synthesis of lipoproteins in the liver, which is one of the earliest, most widespread, and classical inducing toxins in HF models [15]. In live tissue, CYP2E1, cytochrome P4502B1 (CYP2B1), and other cytochromes can convert CCl_4_ to trichloromethyl radicals, which impair the lipid metabolism process of the cell by binding to cellular molecules (nucleic acids, proteins, and lipids), leading to lipoatrophy, which can also react with oxygen to form highly reactive trichloromethyl peroxyl radicals. The attack and destruction of polyunsaturated fatty acids (PUFAs) affect the permeability of mitochondria, the endoplasmic reticulum, and the plasma membrane, resulting in severe damage to cells [16]. Different doses, frequencies, and routes of CCl_4_ administration affect the severity of HF and the survival rate of animals. At present, IP injection, subcutaneous injection, and gavage are the common methods used to administer CCl_4_ in HF models. The IP injection of 50% CCl_4_ in oil solution into mice or rats twice a week for 6–12 weeks usually leads to evident deposition of the collagen matrix in the liver, which is a representative and repeatable HF model [17,18]. Moreover, these in vivo models have been correlated with human liver fibrosis based on immunohistochemical data. For example, in the liver of mice with CCl_4_-induced fibrosis and in fibrotic human livers, immunohistochemistry analysis revealed that the messenger RNA (mRNA) and protein levels of growth differentiation factor 11 (GDF11) are elevated. GDF11 is a member of the transforming growth factor-beta (TGF-β) superfamily, and HSCs are the primary source of GDF11 in the liver [19]. These data related to human HF enhance the reliability of the model for research purposes. CCl_4_ has the advantages of simple operation, low cost, high repeatability, stable pathological characteristics, and a short molding time. However, CCl_4_ is highly toxic, and high-dose injection easily increases the death rate of experimental animals, while the modeling success rate is low with low-dose intervention [20,21].

#### 2.1.3. HF Animal Model Induced by TAA

As early as 1948, the hepatotoxicity of TAA was demonstrated in rodent models [22]. Currently, it has become one of the most frequently used inducers of liver injury and fibrosis. The pathological features of HF are usually simulated by the IP administration of 200 mg/kg TAA to rats 2–3 times per week for 6–8 continuous weeks [23,24]. The mechanism of TAA-induced HF involves affecting mitogen-activated protein kinases (MAPK) and monocyte chemoattractant protein-1 (MCP-1) expression, promoting the downregulation of nuclear factor erythroid 2-related factor 2 (Nrf2), increasing oxidative stress parameters [malondialdehyde (MDA), superoxide dismutase (SOD) and nitrogen monoxide (NO)] and transaminases [alanine aminotransferase (ALT) and aspartate aminotransferase (AST)], and increasing the expression of collagen fibers and vascular endothelial growth factor (VEGF) [25]. TAA-based induction of HF is easily accomplished but has high mortality, as few rodent models can simulate the etiology or pathology of the disease in humans and develop advanced fibrosis. Rats that received 200 mg/kg TAA diluted in 2.2 mL of 0.9% sodium chloride once a week for 24 weeks presented with the appearance of macroscopic lesions of multiple macro- and micronodules with abundant fibrous septa in liver tissue, with elevated levels of AST, ALT, alkaline phosphatase (ALP), and the prothrombin and fibrosis markers alpha-smooth muscle actin (α-SMA), CD68, and type I collagen [26]. This model of advanced chronic fibrosis does not spontaneously reverse at an early stage.

#### 2.1.4. HF Animal Model Induced by DMN

DMN is a deprotonated amine combined with nitroso in a semivolatile oily liquid of the nitrosamine type with hepatic affinity and hepatotoxicity, inducing liver damage and fibrosis with cell necrosis, inflammation, and hemorrhage through methylated proteins [27]. The IP administration of 10 mg/kg DMN to experimental animals for 3 consecutive days per week for 4 weeks results in increased phagocytosis of Kupffer cells in the liver, which are transformed into MFBs and produce large quantities of the ECM [28]. In terms of the molecular characteristics of collagen, DMN-induced collagen is more closely crosslinked than normal, and type III collagen is predominant in early fibrosis [29]. Matrix metalloproteinases (MMPs) are capable of degrading the ECM, whereas tissue inhibitor of metalloproteinases-1 (TIMP-1) is significantly upregulated during DMN-induced liver fibrosis, inhibiting the activity of MMPs and promoting the deposition of the ECM [30]. Joseph et al. revealed that using DMN as an inducer can simulate multiple complications of HF, such as portal hypertension, ascites, and esophageal varices [31]. Compared with other animal models, DMN-induced liver injury is consistent and irreversible, making it suitable for molecular mechanism studies of HF and rapid screening of anti-HF drugs.

Research has suggested that by comparing the histopathological and profibrotic cytokine profiles in HF models constructed using CCl_4_ (1.6 g/kg, for 10 weeks, twice weekly), TAA (200 mg/kg, for 12 weeks, twice weekly), or DMN (10 mg/kg, for 3 weeks, three consecutive days weekly), it was concluded that the progression of HF was more pronounced in DMN- and TAA-induced models and that the most severe deterioration was observed in the DMN-induced model, but its intensely carcinogenic and lethal effects on experimental animals restricted its application [32]. Compared with being induced by CCl_4_, the liver injury in mice was sustained for longer after the last treatment with TAA, which also performed a longer duration of advanced fibrosis [26,33].

### 2.2. Immunological HF Models

Autoimmune hepatitis (AIH) causes inflammatory necrosis of the liver through the attack of immune cells on their own hepatocytes, leading to HF and cirrhosis [34]. Currently, environmental, infectious, or genetic factors contribute to the increased incidence of AIH [35]. Therefore, researchers have used viral infections, concanavalin A (ConA), schistosomiasis, and porcine serum (PS), among other effectors, to mimic AIH fibrosis in an attempt to establish an animal model to determine the potential pathogenesis of AIH.

#### 2.2.1. Virus-Induced HF Animal Models

Macrophages (Mφs) account for 30% of granuloma cells, which produce cytokines and chemokines that mediate HSCs activation and promote collagen synthesis, contributing to the formation of HF [36]. Hepatitis B virus (HBV)-induced Mφs produce immunomodulatory molecules [interleukin-10 (IL-10), TGF-β, and programmed cell death ligand1/2 (PD-L1/2)] that inhibit the antifibrotic effects of natural killer (NK) cells and T cells and secrete cytokines such as platelet-derived growth factor (PDGF) to activate HSCs. Moreover, HBV induces immune-suppressive cells, such as myeloid-derived suppressor cells (MDSCs), NK-regs, and T-regs, to form an immunosuppressive cascade through inhibitory molecules, such as PD-L1, programmed cell death protein 1 (PD-1), and IL-10, which contributes to persistent HBV infection and the progression of HF [37]. Lei Ye et al. successfully used adeno-associated virus serotype 8 (AAV8) to mediate the delivery of the HBV genome to generate a mouse model of hepatitis B, which exhibited increases in ground glass-like hepatocytes and collagen deposition and the upregulation of fibrosis markers [38]. In a study of differentially expressed molecular profiles associated with HBV-mediated HF, the key role of oxidative stress in the progression of HF was revealed, suggesting that catalase (CAT), biliverdin Reductase B (BLVRB), nucleoredoxin (NXN), peroxiredoxin 1 (PRDX1), and isocitrate Dehydrogenase 1 (IDH1) may be potential therapeutic targets for HF [39].

MicroRNA (miRNA) dysregulation during hepatitis C virus (HCV) infection can directly or indirectly affect HCV replication and promote HF by activating the TGF-β signaling pathway, activating HSCs, and increasing the expression of collagen and fibroblast growth factor, which contribute to the development of fibrosis [40]. The nonstructural protein 3-trans-activated protein 1 (NS3TP1) of HCV also promotes the activation, proliferation, and differentiation of HSCs through the TGFβ1/ small mother against decapentaplegic (Smad)3 and nuclear factor-kappaB (NF-κB) signaling pathways to enhance HF, demonstrating that NS3TP1 is a unique new target for the treatment of HF related to HCV [41]. In addition, Du Cheng et al. reported that HCV promotes fibrosis through the tumor necrosis factor alpha (TNF-α) and reactive oxygen species (ROS) -MAPK pathways, consistently activating NF-κB, inducing the secretion of chitinase 3-like protein 1 protein (YKL-40), and promoting the progression of HF through the interaction between HCV and YKL-40, suggesting that the upregulation of YKL-40 may improve the success rate of HF modeling [42]. The above findings suggest that some key targets can be regulated to optimize the HF model and lay the foundation for future research on the therapeutic effects of drugs through these targets. Wenting Li et al. reported that, compared with those subjected to single infection with HBV or HCV, the expression levels of profibrotic genes in co-infected mice further increased, and the underlying mechanism may be related to the regulation of the TGF-β1 signaling-mediated octamer-binding transcription factor 4 (OCT4) and Nanog pathways [43].

Rhesus rotavirus (RRV) is a double-stranded RNA (dsRNA) virus within the *Reoviridae* family that commonly infects newborn mice to induce biliary atresia (BA), a severe hepatobiliary disease clinically characterized by inflammation and progressive fibrosis of the intrahepatic and extrahepatic bile ducts, ultimately leading to cirrhosis and liver failure [44,45]. RRV infection alone cannot successfully induce obvious HF in mice [46]. Therefore, Xinrui Wang et al. [47] developed an HF model in BA mice by the IP injection of 5 μg of anti-Ly6G antibody at 24 h after birth, the IP injection of 20 µL of RRV 4 h later, and the subsequent injection of 10 μg of anti-Ly6G antibody every 3 days for 12 days. A slight increase in collagen deposition in the portal region was subsequently observed at 12 days after RRV injection, and a large amount of collagen deposition was observed on day 42. The levels of ALT, ALP, total bilirubin (TBIL), direct bilirubin (DBIL) and indirect bilirubin (IBIL) were elevated in the livers of the model mice compared with those of the mice with RRV infection alone, suggesting reduced hepatic function in the HF stage of the BA mice. However, only 20–30% of mice entered infection by this integrated modeling method. The time and number of antibodies used are the keys to further exploration to improve the modeling success rate and present an ideal model for studying the mechanism of HF caused by BA.

#### 2.2.2. ConA-Induced HF Animal Models

ConA is a plant lectin that induces immune-mediated liver injury [48]. ConA activates T-cell mitosis and induces leukocyte recruitment and systemic immune activation, and multiple cell types and cytokines interact to trigger HF by inducing cellular inflammation and oxidative stress, which is very familiar with the pathogenesis and histopathological changes in AIH and viral hepatitis in humans [49,50,51]. HF is commonly induced in mice by intravenous injection of ConA (10–20 mg/kg) for 4–8 weeks. In an animal model, the expression levels of type II and IV collagen, α-SMA mRNA, and hydroxyproline (HYP) were elevated in liver tissue, and the serum levels of TGF-β, TNF-α, and TIMP-1 were significantly elevated, whereas matrix metalloproteinase-2 (MMP-2) expression was reduced [52,53]. Jing Zhao et al. [48] experimentally induced acute liver injury by intravenous injection of ConA in wild-type (WT) mice and Toll-like receptor-7 (TLR7)-knockout mice and reported that liver injury was diminished in TLR7-deficient mice, demonstrating that TLR7 modulates the expression of TNF-α in intrahepatic Mφs, exacerbating ConA-induced HF. More research is needed to elucidate the molecular mechanism involved in the progression of ConA-induced liver injury and fibrosis, promoting the screening and discovery of anti-HF drugs for immune-associated hepatitis.

#### 2.2.3. Schistosoma-Induced HF Animal Models

Schistosomiasis is a parasitic disease, the main infection sources of which include *Schistosoma mansoni*, *Schistosoma haematobium,* and *Schistosoma japonicum* [54]. HF model mice are usually percutaneously infected with freshly shed cercariae that are released by *S. japonicum*-infected *Oncomelania hupensis* snails for 4–9 weeks [55,56]. During schistosomal infection, most of the eggs of female worms are deposited in the host liver tissue, triggering inflammation followed by the activation of HSCs into MFBs, leading to HF [57]. In a mouse model infected with *S. japonicum*, severe granulomatous inflammation and tissue fibrosis in the livers, spleens, and large intestines were observed in mice infected for 8 weeks, with a pronounced increase in the number of eosinophils in CD68 macrophage-positive areas; the results indicated that galectin (Gal-1, Gal-3), eosinophils, and Mφs may be essential factors in the process of developing granulomas and fibrosis in *S. japonicum*-infected mice [58]. Xin Qi et al. [59] reported that abundant microRNA-1 (miR-1) produced in Mφ-derived extracellular vesicles (EVs) stimulated by *S. mansoni* soluble egg antigen (SmSEA) could stimulate the autocrine growth of TGF-β3 in HSCs by targeting the inhibition of suppressor of cytokine signaling 3 (SOCS3) expression, thus promoting the activation of HSCs and demonstrating that EVs play an essential role in HSCs activation during the progression of schistosomiasis-associated liver fibrosis (SSLF). Schistosome eggs and SmSEA mediate continuous liver injuries and inflammatory responses, which are the main pathological features of SSLF [60].

#### 2.2.4. Porcine Serum-Induced HF Animal Models

The liver tissue of model rats exhibited massive infiltration of inflammatory cells and large amounts of collagen deposition at 6 weeks after the IP injection of 0.5 mL of PS twice a week, as well as obvious fibrous septa and pseudolobule formation at 9–16 weeks [61]. Elevated β-arrestin2 facilitates collagen production by HSCs and activates the TGF-β1 pathway components Smad2, Smad3, and protein kinase B (AKT), ultimately leading to the formation of HF [61,62]. The model can simulate histopathological changes in human HF disease, such as obvious fibrous septa and pseudolobule formation, but the modeling time is long. In addition, as an allergen, PS causes high mortality in experimental animals during the modeling process [63].

### 2.3. Establishment of HF Animal Models by Surgical Operation

Bile duct ligation (BDL), which can cause cholestatic injury and periportal biliary fibrosis, is the classic method for modeling cholestatic fibrosis with high reproducibility and was initially performed in rats and rabbits. BDL induces severe fibrosis in mice after 21–28 days, with the advantages of low variability and high survival, which are enhanced by optimization under anesthesia, surgical interventions, and posttreatment observations for the dependable reproducibility of the model [64]. The mice underwent BDL to induce cholestasis, increase the expression levels of the inflammatory markers TNF-α and interleukin-1beta (IL-1β), decrease the expression of the anti-inflammatory cytokine IL-10, and trigger inflammation and HF by upregulating TGF-β1/Smad2/α-SMA [65]. The regulation of bile acid plays an important role in cholestatic liver disease. The fibrosis induced after BDL is accompanied by inflammation and collagen deposition, which leads to high mortality, and there is a need to discover a more stable modeling method involving the use of more sophisticated surgical techniques and adhering to the 3R principle for experimental animals [66].

### 2.4. HF Animal Models Induced by Specific Diets

**Figure 1:**
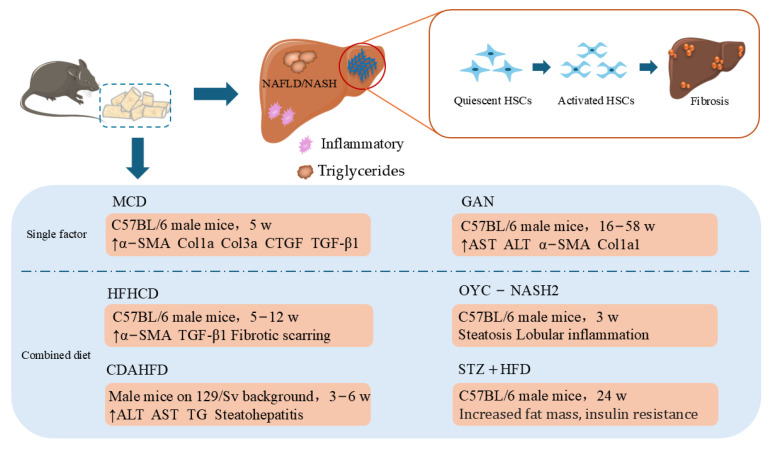
Summary of animal models induced by specific diets for studying HF. Single factor, MCD, GAN; combined diet, HFHCD, CDAHFD, OYC-NASH2, STZ+HFD; NAFLD, nonalcoholic fatty liver disease; NASH, nonalcoholic steatohepatitis; HSCs, hepatic stellate cells; ↑, up-regulated expression.

Nonalcoholic fatty liver disease (NAFLD) affects approximately 25% of the global population [67]. NAFLD manifests as the accumulation of triglycerides (TGs) in the hepatic cytoplasm and encompasses liver diseases such as simple steatosis, nonalcoholic steatohepatitis (NASH), HF, and cirrhosis. NASH, a more severe and progressive form of NAFLD, can present as liver fibrosis or scarring due to inflammation and hepatocyte injury [68,69]. Currently, simple and cost-effective modeling methods, such as a high-fat diet (HFD), methionine choline-deficient (MCD), and Gubra–Amylin NASH (GAN) diet, are widely used to mimic NAFLD/NASH in mice; these methods involve lipid metabolism, inflammation, oxidative stress, and fibrotic pathways through a lack of energy or hepatic lipid content and alter the expression of proteins to achieve the pathological symptoms of HF. However, it is difficult to achieve the pathological process of fibrosis with a single HFD. Moreover, the improvement in and combination of some dietary induction methods also provide more avenues for the therapeutic exploration of HF related to NASH (Figure 1) [70,71].

#### 2.4.1. MCD Diet

As early as 1942, Gyorgy and Goldblatt reported that choline and methionine supplementation had prominent ameliorative effects on liver injury induced by a low-casein diet [72]. Therefore, the MCD diet contains large amounts of sucrose and fat, is deficient in methionine and choline, and is commonly used to induce NASH in animal models. However, some phenotypic characteristics, including obesity and obvious peripheral insulin resistance, are still lacking [73]. MCD-induced hepatic inflammatory cell infiltration and massive lipid accumulation in mice and oxidative stress are mediated by the upregulation of MDA and the downregulation of SOD and glutathione peroxidase (GPX) activities; most notably, the structural disorders of the liver and the accumulation of large amounts of collagen found in the liver after MCD feeding are accompanied by elevated expression levels of profibrogenic genes such as α-SMA, fibrotic marker proteins collagen I and III (COL1A and COL3A), connective tissue growth factor (CTGF) and TGF-β1 [74]. In experiments that typically use 8-week-old C57BL/6J male mice fed an MCD diet for 5 weeks, a significant reduction in body weight and an increase in TG levels with inflammation were detected in the successful establishment of the NASH model and the development of fibrotic features [75,76].

#### 2.4.2. Gubra–Amylin (GAN)-Induced NASH

The GAN NASH diet, which is enriched with 40% saturated fat, 22% fructose, 10% sucrose, and 2% cholesterol, can significantly increase the levels of markers of hepatic lipids, inflammation, and collagen deposition in liver biopsies of obese mice, as revealed by histological and transcriptomic analyses, and most of these changes are consistent with those in patients with NASH [77]. C57BL/6J mice gained weight after 7 weeks of GAN feeding, and the AST and ALT levels increased after 8 weeks but did not change significantly after 16 weeks. Most of the mice developed moderate fibrosis after 16 weeks, and morphological features such as high degrees of steatosis, inflammation, and fibrosis appeared after 28 weeks, significantly increasing the expression levels of α-SMA and Col1a1. Advanced fibrosis occurred after 48 weeks, and reliable progression to tumor development occurred as early as 58 weeks. The above findings suggest that the GAN mouse model better mimics the histopathology, transcription, and metabolism of human HF and has a HF stage, which makes it more appropriate for the study of novel anti-HF drugs [78].

#### 2.4.3. Combined Diet

In general, some specific diets cause only brief steatosis or require longer durations to achieve mild fibrosis. In addition, a Western diet (WD), which is high in fat, fructose (or sucrose), and high-cholesterol (HFC), can mimic the pathogenesis of NASH in humans and induce insulin resistance but does not progress to severe steatohepatitis or advanced fibrosis with prolonged feeding [79]. Consequently, the emergence of multiple combined diets used in conjunction with each other or with other methods has shortened the time to the emergence of fibrosis. For example, compared with HFC or cholic acid (CA) alone, HFC diets containing 2% CA alter the bile acid metabolism and inflammatory responses and induce the development of fibrosis after 9 weeks, with advanced fibrosis appearing at 18 weeks [80,81]. After combined high-fat and HFC (HFHCD) feeding, model mice develop mild fibrosis after 5 weeks, severe fibrotic scarring after 12 weeks, and partial reversal of fibrosis after replacement with a normal diet [82]. In addition, a choline-deficient, l-amino acid-defined, high-fat diet (CDAHFD) could prevent the significant weight loss caused by the MCD diet and induce the development of steatohepatitis, HF, or HCC by impairing the secretion of hepatic very-low-density lipoprotein (VLDL)–TG, with fibrosis usually occurring 3–6 weeks after feeding. This model has the advantages of requiring a short induction period and being cost-effective [83]. In addition, in a study by Qianqian Zheng et al., a model was established that demonstrated significant steatosis, lobular inflammation, and fibrosis after only 3 weeks of feeding, which is a novel model with high reproducibility, versatility, and convenience through a choline-deficient and methionine-restricted high-fat diet (OYC-NASH2) to induce NASH-HCC in mice [84]. Takuma et al. [79] established a mouse model of NASH with advanced fibrosis and the rapid progression of HCC using a HFHCD diet and weekly low-dose IP injections of CCl_4_. The model exhibited histopathological features of human NASH, suggesting that it could be a more efficient experimental tool for preclinical drug trials. Moreover, a high-fat ketogenic diet could increase the accumulation of cholesterol in the liver, which could promote CCl_4_- and TAA-induced fibrosis hallmark expression and hepatic inflammation, exacerbate the severity of HF in mice, and provide more insight into the HF animal model [85].

Recently, the NAFLD mouse model has emerged, which encompasses almost the entire disease progression from TG accumulation to inflammation, hepatocellular injury, HF, and ultimately cirrhosis and/or HCC. The model was established by combining low-dose streptozotocin (STZ) with a HFD administered to C57BL/6J male mice. Notably, NASH and significant fibrosis emerged at 24 weeks. The model is closely associated with the pathological and metabolic features of patients with NAFLD, NASH, and HCC in terms of tissue pathology, metabolic characteristics, and transcriptomics, all of which involve increased fat mass, insulin resistance, dyslipidemia, and visceral adipocyte hypertrophy and inflammation [86]. These studies suggest that the STZ + HFD mouse model may be a suitable choice for preclinical studies of anti-NAFLD drugs.

### 2.5. Genetically Modified HF Animal Models

Genetically modified animal models provide insight into the involvement of specific proteins and signaling pathways in the generation of HF, which could facilitate the identification of potential new drug targets. However, secondary stimulation of compounds such as CCL_4_ can accelerate the progression of HF in genetically modified models [87]. For example, Juan Yang et al. [88] constructed Smad3 gene C-terminal phosphorylation site mutation heterozygote (pSmad3C) mice to increase the relative liver weight, biochemical parameters, collagenous fibers, and fibrotic septa formation. Furthermore, the levels of the fibrosis-related proteins TGF-β1, pSmad2C, pSmad2L, and plasminogen activator inhibitor 1 (PAI-1) are also increased, contributing to fibrogenesis in CCl_4_-induced mice [89]. Typically, monofactorial CCl_4_ induction for less than 4 weeks causes damage to hepatocytes, and continuous administration for more than 8 weeks may lead to cirrhosis [87]. They reported that prolonged 6-week stimulation with CCl_4_ significantly promoted HF under the combined effects of pSmad3C by the gene knock-in technique and microinjection of embryonic stem cells (ESCs). pSmad3C aggravated the relative liver weights, biochemical indices, and formation of collagen fibers and fibrotic septa in CCl_4_-induced HF [88].

Many breakthroughs have been made in the study of HF models using gene knockout technology. According to the research of Zhongyang Lu et al. [90], G protein-coupled receptor 40 (GPR40) knockout in low density lipoprotein receptor (LDLR)-deficient mice may exacerbate HFD-induced hyperlipidemia, hepatic steatosis, inflammation, and fibrosis. GPR40 is a G protein-coupled receptor of free fatty acid (FFA) that can mediate FFA to increase glucose and insulin secretion, playing an important role in the pathogenesis of NASH in T2DM [91,92]. After 57 weeks of HFD feeding, LDLR-deficient mice presented significantly higher cholesterol and FFA levels than normal mice did, revealing the mechanism by which GPR40 KO causes steatosis [90]. The anti-inflammatory effect of GPR40 in Mφs makes it a potential therapeutic target in hyperlipidemia-related NASH. Furthermore, other genetically modified models include ob/ob, db/db, foz/foz, DIAMOND, and MS-NASH transgenic mouse models, and these models require dietary intervention to induce liver damage and fibrosis. For example, ob/ob mice fed an amylin liver NASH (AMLN) diet develop significant hepatic steatosis, inflammation, and fibrosis, which are faster and more severe than those of wild-type mice fed the same diet [93].

To sum up, various types of liver cells, such as immune cells, cholangiocytes, liver sinusoidal endothelial cells (LSECs), and hepatocytes, influence HSCs activation by producing inflammatory and fibrosis cytokines (TGF-β, PDGF, connective tissue growth factor [CTGF], and IL-1β), ROS, and nitric oxide. Because HSCs activation and fibrosis involve multiple cell types, simple in vitro cell culture models cannot fully summarize the course of HF. Therefore, animal models, due to their own complex biological systems, are essential tools for obtaining an in-depth understanding of HF. To study HF with different etiologies, in vivo models induced by chemicals, viruses, cholestasis, specific diets, and gene modifications are commonly used. The induction methods and characteristics of these HF animal models are summarized and presented in Table 1.

## 3. In Vitro HF Models

### 3.1. Primary HSCs

HSCs constitute one of the most important cell types involved in the development of HF, and mesenchymal cells constitute 5–10% of the total hepatocyte population [94]. Quiescent HSCs can be activated and proliferate into MFBs under stimulation with cytokines such as TGF-β1, IL-1, or TNF [95]. The production of collagen fibers is one major activity by which MFBs promote the process of fibrosis. [96]. Primary HSCs derived from human or rodent livers are the classical in vitro models for investigating the pathogenesis of HF. Primary HSCs can be effectively separated from other hepatocytes by density gradient centrifugation, and in addition to being activated under stimulation by profibrotic factors, they can also be cultured on plastic and spontaneously activated in a time-dependent manner, forming MFBs without lipid droplets in 14 days and producing fibrotic components composed mainly of type I and type III collagen and ECM proteins. This process also simulates the deposition of ECM, changes in liver architecture, a decrease in liver elasticity, the influence of blood flow through the liver, and other characteristics in HF, which can more clearly demonstrate the development of HF than the complex mechanisms that explain it in an in vivo model [97,98].

### 3.2. HSCs Lines

In 2005, researchers identified two immortalized HSCs cell lines, LX-1 and LX-2, that provide a more stable and unlimited source of human HSCs, overcoming the problem of low culture reproducibility of primary HSCs. These cell lines are suitable for the study of human HF because they retain key features of cytokine signaling, neuronal gene expression, retinoid metabolism, and fibrogenesis. Like primary HSCs, both cell lines express key receptors regulating HF, including platelet-derived growth factor receptor β (βPDGF-R), obese receptor long form (Ob-R_L_), and discoidin domain receptor 2 (DDR2), and proteins involved in matrix remodeling, such as MMP-2, TIMP-2, and membrane-type I matrix metalloproteinase (MT1-MMP). However, the viability in serum-free medium and the high transfectability of LX-2 cells are unique advantages [99].

In 2013, a novel immortalized murine HSCs line called collagen I(alpha)2-green fluorescent protein (Col-GFP) was generated and characterized. Endoglin modulates TGF-β1 signaling and the differentiation of CFSC-2G cells, an immortalized cell line of rat HSCs origin. This cell line is responsive to profibrogenic stimuli, such as PDGF or TGF-β1, and can activate intracellular signaling pathways such as Smads and MAPK. Moreover, increased phosphorylation of ERK1/2 and upregulation of vimentin (VIM), α-SMA, and connective tissue growth factor indicate that this CFSC-2G cell line with an HSCs origin is considered a novel experimental tool with different fibrotic properties [100]. In 2015, two portal myofibroblast lines, RGF and RGF-N2, were isolated from adult rat livers. Both cell lines expressed myofibroblast markers such as α-SMA, TIMP-1, and type XV collagen alpha-1 but lacked expression of the HSCs-specific markers desmin and lecithin retinol acyltransferase. These two cell lines represent a resource for portal myofibroblast studies and for the study of novel in vitro models of HF [101].

In fact, the communication between various cells in liver tissue and HSCs is crucial for the development of HF. Hence, some coculture methods, such as hepatocyte and HSCs coculture [102], blast cell and primary HSCs coculture [103], and endothelial cell and HSCs coculture [104], have been performed with HSCs of different cell lines. In the future, the conditions for the establishment of these 2D coculture systems should be continuously optimized, and techniques such as transcriptomics and metabolomics can be used to explore the molecular mechanisms of the cell–cell interactions.

### 3.3. Three-Dimensional In Vitro HF Model

Currently, there is a growing number of studies on 3D in vitro modeling methods for identifying more complex cytological mechanisms involved in HF. These methods are primarily divided into applications such as 3D cocultures, 3D liver organoids, 3D-bioprinted liver tissues, and precision-cut liver slice culture (PCLS).

#### 3.3.1. Three-Dimensional Coculture Models

Three-dimensional coculture liver models allow better prediction of drug efficacy and toxicity in humans, as well as the ability to predict the metabolism and clearance of drug candidates or marketed drugs. A series of 3D coculture models that simulate HF have been developed in recent years. For example, Mannaerts et al. [105] established a cocultured spheroid with freshly isolated mouse hepatocytes (HEPs) and HSCs. The hybrid spheroids better reflected liver injury-dependent HSCs activation and the related markers actin alpha 2 (Acta2), Col1a1, Col3a1 and lysyl Oxidase (LOX). The genes with in vivo-like profiles in the spheroids included ECM contributing genes such as microfibril-associated glycoprotein 4 (Mfap4), collagen VI-alpha3 chain (Col6a3), and fibromodulin (Fmod), which promote the proliferation, migration, and invasion of HSCs, contributing to their fibrogenic activity. Thus, 3D cocultures better reproduce HSCs than commonly used 2D monocultures do. Similarly, Ensieh et al. [106] used micropatterning to develop an HSCs-activated spheroid composed of 3D coculture microtissues composed of HepaRG cells and human umbilical vein endothelial cells (HUVECs) and cocultured it with inactivated or activated LX-2 cells to study the role of aHSCs in the process of HF. The results showed that aHSCs may lead to epithelial-to-mesenchymal transition (EMT) of hepatocytes during HF, mainly through activation of the TGF-β/Smad signaling pathway, reflecting the importance of effective ex vivo models of 3D liver microtissues for exploring the mechanisms of antifibrotic components. In addition, Ho-Joon Lee et al. [107] established a new human HSCs line (LSC-1), which was applied to diverse types of three-dimensional (3D) coculture systems involving differentiated HepaRG cells, characterized by various HSCs markers, including glial fibrillary acidic protein (GFAP), VIM, nestin, endoglin, PDGFR-β, and α-SMA. Compared with conventional monolayer cultures, LSC-1 cultures presented the upregulation of fibrogenic genes and increased levels of matrix and adhesion proteins. They reported that LSC-1 not only supports the liver function and differentiation status of cocultured hepatocytes but also enhances TGF-β1 responsiveness through its distinct ECM network. Moreover, compared with LX-2 cells, TGF-β1-treated cells are more responsive; thus, this new model provides another alternative for future models of liver fibrosis. In brief, spheroid cultures of primary human hepatocytes (PHHs) and primary HSCs, LSECs, and Kupffer cells are good models of HF in vitro. The relatively small number of cells (±2000) required to establish such spheroids does not preclude the use of a limited number of PHHs. However, these cultures have several drawbacks, such as the more cumbersome nature of handling relatively small (<200 μm) spheroids [108].

#### 3.3.2. Three-Dimensional Organoid Models

In the 1980s, the first pluripotent stem cells (PSCs) were successfully isolated from mouse embryos; subsequently, mesenchymal stem cells (MSCs), human ESCs, and induced PSCs (iPSCs) were obtained, and these developments and advances in stem cell technology laid the foundation for research in the organoid field [109]. Three-dimensional organoids are cultures of stem cells and tissues that develop and grow from ESCs in vitro or from adult stem cells, with cell type complexity that structurally and functionally mimics human organs better than 2D cultures do [110]. In 2013, leucine-rich-repeat-containing G-protein-coupled receptor 5 (LGR5) progenitor-like oval cells that appeared in the portal triad region after liver injury in mice expanded into transplantable organoids under the Wnt-driven regenerative response and resembled the adult liver [111]. By 2018, more mature and durable hepatocyte organoids were established from purified axis inhibition protein 2 (Axin2) mouse hepatocytes [112]. After years of research in the field of hepatocyte organoids, these organoids have been used to study hepatopathies such as Alagille syndrome, fatty liver disease, Wilson disease, HBV infection, and cystic fibrosis [113].

Seon et al. [114] developed novel human pluripotent stem cell-derived hepatocyte-like liver organoids, which are characterized by self-renewal and are morphologically indistinguishable from epithelial organoids derived from adult liver tissue. These organoids preserve mature liver properties, including serum protein production, drug metabolism and detoxifying functions, active mitochondrial bioenergetics, and regenerative and inflammatory responses. Therefore, these organoids help to better demonstrate liver development and regeneration, outlining the phenotypes of human liver disease. In addition, Rie Ouchi et al. [115] derived a multicellular human liver organoid (HLO) composed of hepatocytes and stellate and Kupffer-like cells and simulated the fibrotic response in the HLO by FFA exposure. HLO showed lipid accumulation in a dose-dependent manner with oleic acid (OA), and this FFA-treated HLO was defined as steatohepatitis HLO. Within 7 days of OA treatment, HLO increased VIM and α-SMA expression and collagen deposition, and elevated procollagen type III N-terminal peptide (P3NP), a marker of liver fibrosis, reflected the development of fibrosis-like pathology in this organ model by persistent FFA exposure.

The development of organoid technology is extremely important for the study of HF. Organoids can mimic key cell types and structural features of the organ, have a high degree of genetic stability, maintain cellular phenotypes, and are suitable for the study of disease mechanisms. However, there are limitations to the development of liver organoids, such as the challenges of hepatocyte maturation, culture longevity, and large-scale production of pure cultures [113].

#### 3.3.3. Three-Dimensional-Bioprinted Liver Tissues

Three-dimensional bioprinting is a promising and innovative biofabrication technology for building artificial multicellular tissues or organs by combining biologics with 3D structures [116]. Three-dimensional-bioprinted human liver tissues can consist of primary human parenchymal (hepatocyte) and nonparenchymal (endothelial and hepatic stellate) cell populations [117]. Xuanyi Ma et al. [118] developed a photocrosslinkable liver decellularized extracellular matrix (dECM) and a rapid light-based 3D bioprinting process that can be easily printed into liver lobule architectures and has a biomimetic fibrous septa design, laying a solid foundation for the construction of more complex HF or cirrhosis disease models. Leah M Norona et al. [119] demonstrated the utility of bioprinted tissue constructs composed of primary hepatocytes, HSCs, and endothelial cells in simulating fibrosis from methotrexate (MTX)- and TAA-induced liver injury. This simulated fibrosis is characterized by the deposition and accumulation of fibrillar collagens similar to those observed in clinical samples obtained from patients with HF, and the upregulation of the fibrosis-associated genes ACTA2 and COL1A1 mimics classical wound-healing responses. These data confirm that 3D-bioprinted liver tissue can be used to visualize drug-, chemical-, and TGF-β1-induced fibrogenesis at the cellular, molecular, and tissue levels. In recent years, by combining cellular, bioprinting, and microfluidic technologies, biomaterials have better mimicked the disease microenvironment, and the combination of these technologies has helped to replicate complex tissue structures, which will contribute greatly to disease modeling, drug discovery, and even regenerative medicine [120]. However, the limitations of diverse preparation techniques cannot be ignored, and 3D-printed liver tissue remains a biological strategy that deserves more research.

#### 3.3.4. Precision-Cut Liver Slice Culture

Precision-cut liver slice culture (PCLS) is an alternative to 3D ex vivo HF modeling. This versatile ex vivo model mimics the multicellular organization of the liver environment and is able to maintain the viability of hepatocytes, Kupffer cells, endothelial cells, and HSCs, and the liver sections are reproducible and inexpensive [121]. PCLS requires the use of instruments and equipment such as a tissue slicer, mechanical drill, and incubation cabinet to process sectioned liver tissues (250 μm) derived from animals and humans, and the liver tissue sections produced in PCLS activate HSCs within 48 h, leading to liver fibrosis [122]. Una et al. [123] compared the extent of hepatocyte death induced by treatment with ethanol alone or in combination with fatty acids and lipopolysaccharides (FA + LPS) in PCLS and reported profibrotic activation in sections (i.e., TIMP-1, TGF-β1, COL1A1, and PDGFRB were significantly upregulated). Notably, the use of mechanical cutting to prepare PCLS induces profibrogenic wound-healing activity, and these results highlight the versatility of PCLS and its applicability in drug discovery. Liza et al. [124] improved PCLS by supplementing 3 mm mouse PCLS cultures with valproate to significantly reduce fibrosis and increase cell viability for up to 5 days, and fibrosis could be induced by other compounds, such as acetaminophen, allowing application of the improved PCLS to be used with antifibrosis compounds.

In summary, as shown in Table 2, various in vitro models have been established to explain the molecular mechanisms of multicellular involvement in the progression of HF, and some cell HF models based on organoids and gene editing should be further developed for antifibrosis drug discovery.

## 4. Conclusions and Perspectives

HF is a reversible process, and early treatment can inhibit the progression of fibrosis or even cause its regression; therefore, the use of in vivo and in vitro models of HF to study therapeutic strategies and potential targets for HF has been a hot topic. An increasing number of studies on HF require experimental models with stable modeling methods, high modeling rates, good reproducibility, and increased efficiency and closer resemblance to the pathological features of human HF.

To date, researchers have employed diverse experimental animal and in vivo and in vitro modeling approaches to simulate complex hepatic cell–cell interactions and signaling pathways, steatosis, inflammation, oxidative stress, and decompensation characteristics of human HF. Consequently, numerous commonly utilized HF models have been successfully developed. Nevertheless, due to the genetic background disparities between humans and other animal species, conventional single-cell culture models are incapable of comprehensively encompassing the multicellular and multidirectional evolution of human fibrosis. Moreover, animal models of HF have been demonstrated to only partially reflect human diseases [125,126]. In recent years, an increasing number of preclinical investigations and validation guidelines for potential antifibrotic drugs have been proposed, and both the commonly adopted liver fibrosis modeling methods and the newly developed 3D modeling approaches have become increasingly mature. It is proposed that antifibrotic drugs be investigated in the combined utilization of validated complementary fibrosis models, considering the complex pathophysiology of the disease, complementary cell culture models, and the microenvironment of 3D modeling, which might facilitate future antifibrotic opportunities.

Importantly, there is no perfect HF disease model, either in vivo or in vitro, and the generation of novel HF models will continue. In the present review, we summarize currently available in vivo and in vitro models to provide researchers with a more accurate way to answer research questions and establish a foundation for the development of additional model combinations that can help researchers explore new mechanisms of HF and test new drugs before they enter clinical trials.

## Figures and Tables

**Table 1:** In vivo animal models of HF.

Inducements	Species	Methods	Duration	Characteristics	Reference
Ethanol	/	Mixed with water or liquid diets, Intragastric infusion	/	ROS ↑, HSCs ↑	[9,10,11,12]
CCl_4_	Male SD rats	IP injection, Subcutaneous injection (50%, made with olive oil, 2 mL/kg)	6–12 w	Kupffer cells ↑, HSCs ↑, ECM ↑	[15,16,17,18,19]
TAA	Male SD rats	IP injection (200 mg/kg, 3 times/week)	6–8 w	MDA ↑, SOD ↑, NO ↑, ALT ↑, AST ↑, ALP ↑, Nrf2 ↓	[23,24,25,26]
DMN	Male Wistar rats	IP injection(10 mg/mL, 10 mg/kg)	4 w	Kupffer cells ↑, ECM ↑, III collagen ↑	[28,29,30,31]
HBV	C57BL/6 mice	Tail vein injection	4–5 w	Ground glass-like hepatocytes ↑, collagen deposition ↑	[37,38,39]
HCV	C57BL/6 mice	Tail vein	/	TGF-β ↑, HSCs ↑, TNF-α, ROS-MAPK ↑	[40,41,42,43]
RRV	BALB/c mice	IP injection (20 μL, titer 1.5 × 10^6^ PFU/mL)	2–6 w	ALT ↑ALP ↑, TBIL ↑, DBIL ↑, IBIL ↑	[44,45,46,47]
ConA	Wild-type male C57BL/6 mice	Intravenous injection (10–20 mg/kg)	4–8 w	T-cell mitosis ↑, II and IV collagens ↑, α-SMA mRNA, HP, TGF-β ↑, TNF-α ↑, TIMP-1 ↑, MMP-2 ↓	[48,49,50,51,52,53]
Schistosoma	Female C57BL/6 mice	Percutaneous infection (*S. japonicum*-infected *Oncomelania hupensis* snails)	4–9 w, 8 w (severe)	EVs ↑miR-1 ↑, SOCS3 ↓, HSCs ↑, TGF-β3 ↑	[55,56,57,58,59]
PS	Male SD rats	IP injection (0.5 mL, twice weekly)	12–16 w	HSCs ↑ECM ↑, β-arrestin2 ↑, TGF-β ↑, TβRⅢ ↓, collagen I and collagen III ↑	[61,62,63]
BDL	Male C57BL/6 wild-type mice	Double ligation of the common bile duct	3–4 w	TGF-β1/Smad2/α-SMA ↑, TNF-α ↑, IL-1β ↑	[64,65,66]
MCD	C57BL/6 male mice	Feeding (methionine- and choline-sufficient diet)	5 w	MDA ↑SOD ↓, GPX ↓, α-SMA ↑, Col1a ↑, Col3a ↑, CTGF ↑, TGF-β1 ↑	[72,73,74,75,76]
GAN	C57BL/6 J mice	Feeding (4.49 kcal/g, 40 kcal-% fat, 22% fructose, 10% sucrose, 2% cholesterol)	16–58 w	AST ↑, ALT ↑, α-SMA ↑, Col1a1 ↑	[77,78]
HFHCD	C57BL/6J male mice	Feeding (HFD supplemented with cholesterol and containing a high percentage of saturated fatty acids)	5–12 w	Fibrotic scarring ↑	[82]
CDAHFD	Male mice on 129/Sv background	Feeding (18% protein, 36% carbohydrate, 46% fat)	3–6 w	TG ↑, Steatohepatitis	[83]
OYC-NASH2	C57BL/6J male mice	Feeding (choline-deficient and methionine-restricted HFD)	3 w	Steatosis ↑, TGF-β1 ↑, IL-1β ↑	[84]
STZ + HFD	C57BL/6J male mice	IP injection (low-dose STZ) + Feeding(HFD)	24 w	Fat mass ↑	[86]
pSmad3C	C57BL/6J mice (WT, genotype: pSmad3C^+/+^)	Genetically modified mice	6 w	TGF-β, Smad3 ↑, TGF-β1 ↑, pSmad2C ↑, pSmad2L ↑, PAI-1 ↑	[87,88,89]
GPR40 knockout	LDLR-deficient mice on a C57BL/6 background	Genetically modified mice	16 w	Mediate FFA ↑, Glucose and insulin secretion ↑	[87,90,91]

↑, up-regulated expression; ↓, down-regulated expression.

**Table 2:** In vitro models of HF.

Models	Inducers	Duration (Fibrosis)	Characteristics	Reference
Primary HSCs	TGF-β1, IL-1, TNF	/	TNF ↑, vitamin A ↓, PPARγ ↓, ECM ↑, α-SMA ↑	[94,95,96,97]
HSCs lines	SV40 T antigen, Fetal bovine serum	/	βPDGF-R ↑, Ob-R_L_ ↑, DDR2 ↑, MMP-2 ↑, TIMP-2 ↑, MT1-MMP ↑, α-SMA ↑	[98,99,100,101,102,103]
HEP–HSCs 3D coculture	2 mM APAP	72 h	Acta2 ↑, Col3a1 ↑, Col1a1 ↑, Lox ↑, TGF-β1 ↑	[105]
HepaRG–HUVEC 3D coculture	LX-2 cells, TGFβ-1	/	TGF-β/SMAD, EMT, Acta2 ↑, Col1a1 ↑, MMP2 ↑, TIMP1 ↑	[106]
LSC-1–HepaRG 3D coculture	SV-40, hTERT, Fibrinogen–thrombin	3 w	Col4A3 ↑, LAMb1 ↑, CYP2B6 ↑, 3A4 ↑, Type IV collagen ↑, α-SMA ↑, Col1A1 ↑	[107]
3D organoid	HLO+ FFA	1 w	VIM ↑, α-SMA ↑, P3NP ↑	[108,109,110,111,112]
3D-bioprinted liver tissues	TGF-β1, MTX, TAA	2 w	ACTA2 ↑, COL1A1 ↑, Collagen I ↑, Collagen IV ↑, α-SMA ↑	[115,116,117,118,119]
PCLS	TGF-β1, APAP	48 h	Col1a1 ↑, Col5a2 ↑, Lox ↑, TIMP-1, TGF-β1, COL1A1, and PDGFRB ↑	[120,121,122,123]

↑, up-regulated expression; ↓, down-regulated expression.
